# Biochemical characterization of trans-sialidase TS1 variants from *Trypanosoma congolense*

**DOI:** 10.1186/1471-2091-12-39

**Published:** 2011-07-30

**Authors:** Hendrik Koliwer-Brandl, Thaddeus T Gbem, Mario Waespy, Olga Reichert, Philipp Mandel, Eric Drebitz, Frank Dietz, Sørge Kelm

**Affiliations:** 1 Centre for Biomolecular Interactions Bremen, Department of Biology and Chemistry, University of Bremen, Leobener Str./NW2/B2235, 28359 Bremen, Germany

## Abstract

**Background:**

Animal African trypanosomiasis, sleeping sickness in humans and Nagana in cattle, is a resurgent disease in Africa caused by *Trypanosoma *parasites. Trans-sialidases expressed by trypanosomes play an important role in the infection cycle of insects and mammals. Whereas trans-sialidases of other trypanosomes like the American *T. cruzi *are well investigated, relatively little research has been done on these enzymes of *T. congolense*.

**Results:**

Based on a partial sequence and an open reading frame in the WTSI database, DNA sequences encoding for eleven *T. congolense *trans-sialidase 1 variants with 96.3% overall amino acid identity were amplified. Trans-sialidase 1 variants were expressed as recombinant proteins, isolated and assayed for trans-sialylation activity. The purified proteins produced α2,3-sialyllactose from lactose by desialylating fetuin, clearly demonstrating their trans-sialidase activity. Using an HPLC-based assay, substrate specificities and kinetic parameters of two variants were characterized in detail indicating differences in substrate specificities for lactose, fetuin and synthetic substrates. Both enzymes were able to sialylate asialofetuin to an extent, which was sufficient to reconstitute binding sites for Siglec-4. A mass spectrometric analysis of the sialylation pattern of glycopeptides from fetuin revealed clear but generally similar changes in the sialylation pattern of the *N*-glycans on fetuin catalyzed by the trans-sialidases investigated.

**Conclusions:**

The identification and characterization of a trans-sialidase gene family of the African parasite *T. congolense *has opened new perspectives for investigating the biological role of these enzymes in Nagana and sleeping sickness. Based on this study it will be interesting to address the expression pattern of these genes and their activities in the different stages of the parasite in its infection cycle. Furthermore, these trans-sialidases have the biotechnological potential to be used for enzymatic modification of sialylated glycoconjugates.

## Background

Animal African trypanosomiasis, called sleeping sickness in humans and Nagana in cattle, is a resurgent disease in Africa. Nagana is caused by *Trypanosoma congolense *(*T. congolense*), *Trypanosoma vivax *(*T. vivax*) and *Trypanosoma brucei *(*T. brucei*) subspecies. Most research on African trypanosomes has focused on *T. brucei*, whereas only few studies have been done with other African trypanosomes including *T. congolense*. In wild animals, these parasites cause relatively mild infections while in domestic animals they cause a severe, often fatal disease. Because of Nagana, stock farming is very difficult within the tsetse belt of Africa [1].

Although of crucial importance for their survival, cyclical transmission and hence pathogenicity of trypanosomes, trypanosomes lack the biochemical metabolic machinery synthesizing sialic acids (Sia), but use a unique enzyme, trans-sialidase (TS) to transfer Sia onto the parasites surface molecules from the environment. Structurally TS belong to the family of sialidases (SA). In contrast to the usual sialyltransferases, TS does not utilize CMP-activated Sia as monosaccharide donors, but catalyzes the transfer of carbohydrate-linked Sia to another glycan forming a new α2,3-glycosidic linkage to galactose or *N*-acetylgalactosamine.

Whereas more detailed studies exist on the role of TS in the pathogenicity of *T. cruzi*, the etiologic agent of Chagas diseases in South America, where TS was first discovered [2], the current knowledge about the corresponding enzymes in the African trypanosomes is very limited. Of all the African trypanosomes, only *T. brucei *full length TS genes have been cloned and studied [3]. Sialylation of parasite surfaces is believed to protect the parasites from the action of glycolytic enzymes as well as from immunocompetent substances present in the tsetse gut and blood meal respectively, as well as influencing the interaction of parasites with the gut epithelial cells [4-6]. In the African trypanosomes where TS is thought to be expressed only in the procyclic insect stages [5,7], TS has been shown to increase the survival, maturation and hence establishment of the parasites in the vector midgut [8].

Two TS forms, named TS-form 1 and TS-form 2, have been purified from procyclic *T. congolense *cultures [6]. Interestingly, glutamic acid and alanine-rich protein (GARP) was co-purified with TS-form 1, suggesting that GARP may be a natural substrate for TS-form 1. Interestingly, TS-form 1 had significantly less SA activity and higher TS activity, whereas SA activity was predominately found in preparations of TS-form 2. An anti-*T. congolense *TS antibody (mAb 7/23) was developed using TS form 1 as antigen. This antibody is specific for *T. congolense *TS recognizing TS-form 1 and TS-form 2, but does not bind to *T. brucei *TS. Peptides micro sequencing revealed the amino acid sequence VVDPTVVAK in TS-form 1. Subsequently, fragments of two TS genes (TS1 and TS2) were sequenced, sharing about 50% sequence identity [9]. TS1 encoded this peptide sequence, whereas in TS2 this sequence ended in VVK. These data strongly suggested that the gene product of TS1 has been present in TS-form 1. Nevertheless, it has remained unclear whether only TS1 and/or TS2 gene products were present in TS-form 1 and TS-form 2 preparations. Due to the fact that the monoclonal antibody mAb 7/23 bound both TS preparations, it is quite possible that TS-form 2 contained at least some amounts of TS1 gene product, which might have been responsible for the TS activity of this preparation.

Here, we report the cloning of eleven trans-sialidase TS1 variants from *T. congolense *and their recombinant expression in CHO_Lec1 _cells. Furthermore, the enzymatic properties of two of these recombinant TS1 variants were compared with TS from *T. cruzi*.

## Results

### Diversity of TS1 genes and structural model

**Figure 1:**
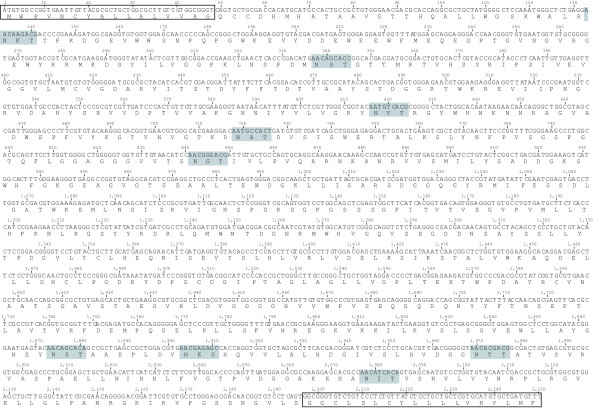
**Primary sequence of TS1a**. The full length coding domain sequence [EMBL: HE583283] with corresponding amino acid translations is shown. The recombinant protein was generated without the N-terminal signal peptide and without the C-terminal region predicted to be replaced by a GPI anchor in the native protein (framed boxes). Predicted *N*-glycosylation sites are highlighted by light grey boxes.

Based on the partial sequence of TS1 [GenBank: AJ535487.1] [9], an open reading frame in the Welcome Trust Sanger Institute (WTSI) database was identified. The full-length translation product consists of 750 amino acids extending the partial sequence of TS1 by 153 amino acids at the N-terminus and by 84 amino acids at the C-terminus. It contains a 16 amino acids N-terminal signal peptide and a catalytic domain (residues 17-467), which is connected through a long α helix (residues 468-491) to a lectin domain (residues 492-732) followed by a potential C-terminal GPI-anchor attachment site (residues 733-750, identified by big-PI predictor [10]). Furthermore, nine potential *N*-glycosylation sites were identified (Figure 1).

**Figure 2:**
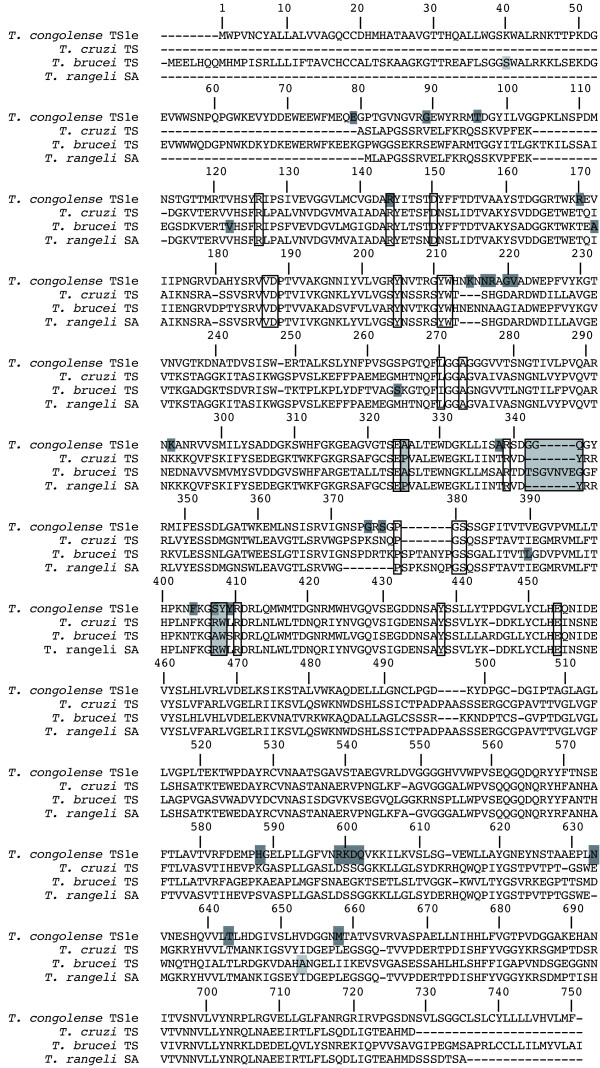
**Primary sequence alignment of trypanosomal trans-sialidases**. The amino acid sequence of *T. congolense *[EMBL: HE583287] TS1 e-1 was aligned with *T. cruzi *TS [EMBL: BAA09334.1, PDB: 3B69], *T. brucei *TS [EMBL: AAG32055.1] and *T. rangeli *SA [EMBL: AAC95493.1] based on a structural alignment of *T. congolense *TS1 e-1 with *T. cruzi *TS generated with Yasara during homology modeling. Amino acids, which have been proposed to be relevant for enzymatic activity are marked with black frames if conserved or with black frames and light grey background if not conserved. Positions with variations occurring in *T. congolense *TS1 or *T. brucei *TS [3] are highlighted by dark grey boxes.

*T. congolense *TS1 shares about 57% sequence identity with *T. brucei *TS [EMBL: AAG32055.1] and 48% with *T. cruzi *TS [EMBL: BAA09334.1] (Figure 2). The *T. brucei *TS has a prolonged N-terminus of approx. 90 amino acids, which is conserved in *T. congolense *TS1 sharing 50% amino acids, but is absent in *T. cruzi *TS. The catalytic domain of both African proteins has 60% and the lectin domain 43% sequence similarities. *T. congolense *TS1, like *T. brucei *TS and *T. rangeli *SA, has no C-terminal SAPA domain typical for *T. cruzi *TS [11]. Almost all amino acid residues reported to be required for TS activity are identical in TS1 with the exception of A325 (corresponding to P231 in *T. cruzi *TS) [11], R127, G344-Q346 and Y408 (corresponding to Y248 and W312, respectively in *T. cruzi *TS) [12] (Figure 2).

**Figure 3:**
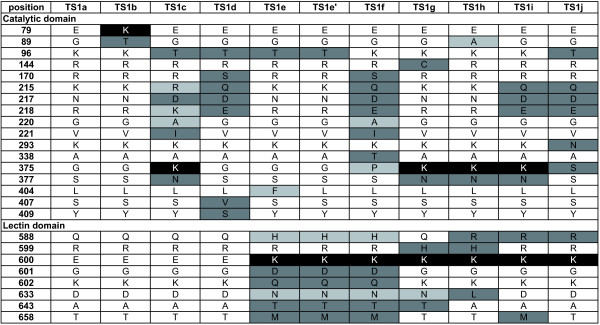
**Amino acid variations found in *T. congolense *TS1a-TS1j**. TS1a [EMBL: HE583283], TS1b [EMBL: HE583284], TS1c [EMBL: HE583285], TS1d [EMBL: HE583286], TS1 e-1 [EMBL: HE583287] , TS1 e-2 [EMBL: HE583288], TS1f [EMBL: HE583289], TS1g [EMBL: HE583290], TS1h [EMBL: HE583291], TS1i [EMBL: HE583292], TS1j [EMBL: HE583293]. Differences in amino acids are highlighted (light grey: conservative; dark grey: modest; black: drastic change).

**Figure 4:**
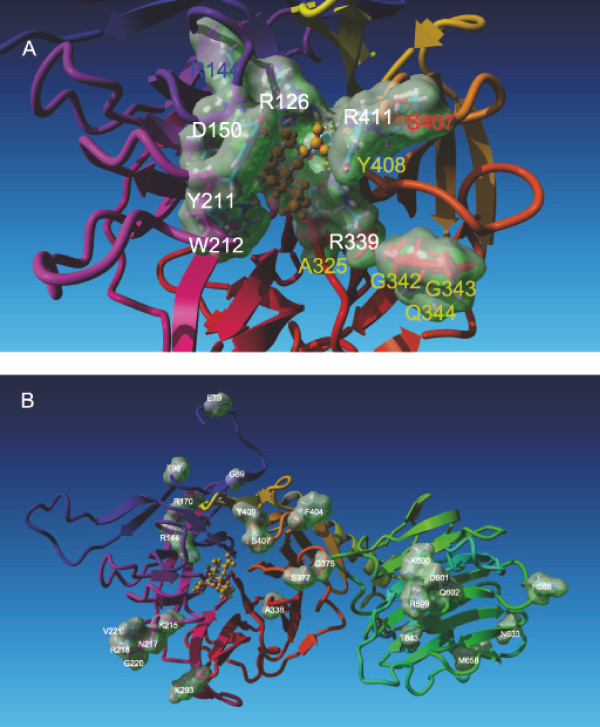
**Homology model of *T. congolense *TS1**. The crystal structure of *T. cruzi *TS [12] in complex with 3-fluoro-5-*N*-acetyl-9-benzamido-2,9-dideoxy-neuraminic acid was used as template to calculate a model structure for *T. congolense *TS1 e-1. Only the Neu5Ac part (orange) of the ligand in the binding site of the complex is illustrated. (A) Conserved amino acids of the active site are labeled in white. Amino acids at positions reported to be important for ligand binding in *T. cruzi *TS [12], which are not conserved in *T. congolense *are labeled in yellow. The red labeled position 407 is a serine or valine in *T. congolense *TS1 forms. R144, which is exchanged to a cysteine in TS1g, is labeled in blue. (B) Green clouds mark positions at which different amino acids occur in other *T. congolense *TS1 forms.

To produce recombinant protein for enzyme characterization, the DNA encoding amino acids 17-732 was amplified using genomic *T. congolense *DNA as a template and inserted into a mammalian expression vector as described in Methods. 13 clones were picked from two independent cloning experiments and sequenced. Interestingly, not all the 13 clones had identical sequences and eleven different sequences were obtained (TS1a through TS1j), exhibiting an overall amino acid identity of 96.3%. A more detailed search of the WTSI database using these sequences as queries confirmed the presence of these TS1 genes in the *T. congolense *genome database. As shown in Figure 3, changes are not evenly distributed over the protein sequences. Eight were found in the lectin domain and 17 in the catalytic domain, some close to the predicted active site as shown in Figure 4A.

For a better understanding of how these differences may affect TS function, we calculated a model structure (Figure 4) for TS1 e-1 by homology modeling based on the crystal structure of *T. cruzi *TS [12], which was complexed with the Sia derivative 3-fluoro-5-*N*-acetyl-9-benzamido-2,9-dideoxy-neuraminic acid. The superimposed structures of *T. cruzi *TS and the *T. congolense *TS1 e-1 model had a root mean square deviation (RMSD) of 0.685 Å over 594 aligned residues.

In Figure 4A, amino acids of the active site are highlighted. Most of the amino acids reported to be relevant for TS activity are identical in all *T. congolense *TS1 variants (white labels). However, differences to *T. cruzi *TS were identified at three positions (yellow labels in Figure 4A). (I) At position 325 all *T. congolense *TS1 variants have an alanine, like in *T. brucei *TS, replacing a proline occurring in *T. cruzi *TS (P231); (II) Y408 of all *T. congolense *TS1 variants corresponds to a tryptophan in *T. cruzi *TS (W321) and *T. brucei *TS; (III) the group of G342, G343 and Q344 replaces a tyrosine (Y248) in *T. cruzi *TS. In addition, near the catalytic site at position 407 (red label) in *T. congolense *TS1 variants, a serine or valine occurs instead of arginine (R311) in *T. cruzi *TS. Interestingly, similar differences occur also in *T. brucei *TS (Figure 2). Since these amino acids are close to the active site, they could influence the acceptor binding specificity. The arginine at position 144 (blue label) is conserved in all TS, with the exception of *T. congolense *TS1g, where it is a cysteine.

In Figure 4B the amino acid positions are highlighted, which have different side chains in TS1a-TS1j (Figure 3). It should be noted that these are all on the same side of the protein as the catalytic site. Striking is a cluster of amino acid variations in the lectin domain (position 599 to 602 and 643) suggesting that these changes may influence substrate binding of larger substrate molecules, such as glycoproteins.

### Characterization of *T. congolense *TS1 enzyme activity

All eleven TS1 gene products (TS1a-TS1j) were expressed as recombinant proteins and were recognized by the anti- *T. congolense *TS antibody (mAb 7/23) [6] (data not shown). For all TS1 variants similar robust TS activity could be determined, except for TS1g. This variant, which carries cysteine instead of arginine at position 144, had only very low TS activity. However, in contrast to the other variants, TS1g released free Sia from fetuin at about 50% of the transfer to lactose. Two of the *T. congolense *TS1 variants, TS1b and TS1 e-1, were further characterized. They differ in eleven of the total 25 positions with amino acid variations listed in Figure 3, three in the catalytic domain and eight in the lectin domain.

**Figure 5:**
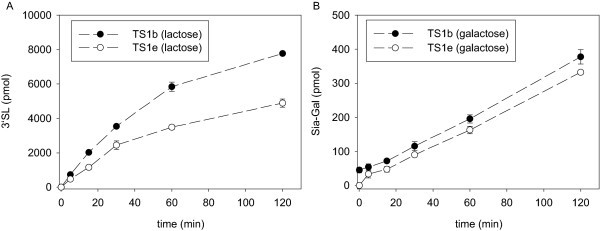
**Time dependence of TS reactions**. Reactions of 100 μg fetuin with 100 nmol lactose (A) or 100 nmol galactose (B) were started with 50 ng TS in 50 μL and incubated at 37°C. The amount of 3'SL produced was determined by HPAEC-PAD as described under Methods. Data points are means ± standard deviations of three replicates.

The donor substrates fetuin 3'SL or pNP-Neu5Ac and the acceptor substrates lactose, galactose or Gal-MU were employed to determine sialidase and trans-sialidase activities. For this purpose, a new assay was established as described under Methods, using HPAEC-PAD to quantify sialylated oligosaccharide products with the detection limit of 20 pmol 3'SL corresponding to 0.5 μM in the reaction mixture. In standard assays, 50 μL TS reactions were set up with 50 ng TS1b or TS1 e-1, 100 μg fetuin (approx. 600 μM bound Sia) as donor substrate and 100 nmol acceptor substrate (2 mM e. g. lactose or galactose). Under these conditions, linear product formation was obtained for up to 2500 pmol corresponding to 50 μM 3'SL (Figure 5).

If lactose is used as a donor substrate under standard conditions, 3'SL concentration increases linearly for about 30 minutes before the reaction velocity started to decrease (Figure 5A). It should be noted that lactose was sialylated almost twice as fast by TS1b than by TS1 e-1. In contrast to lactose, galactose was sialylated at the same rate by both enzymes, but at about 20-fold lower velocity than lactose.

**Figure 6:**
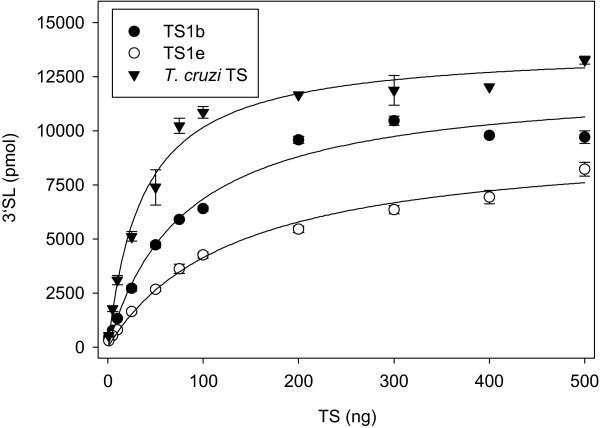
**Concentration dependence of TS reactions**. Reactions of 100 μg fetuin with 100 nmol lactose were started with varying amounts of TS in overall 50 μL 10 mM phosphate buffer, pH 7.4 and incubated at 37°C for 30 min. The amount of 3'SL produced was determined by HPAEC-PAD as described under Methods. Data points are means ± standard deviations of three determinations.

Different specific activities were obtained for *T. congolense *TS1b, TS1 e-1 and *T. cruzi *TS (Figure 6). The reaction velocity was linearly dependent on the amount of TS as long as the concentration of the product 3'SL was below 50 μM. Under standard conditions 50 μM 3'SL was produced in 30 minutes with 50 ng TS. If 200 ng TS or more were used, product formation was independent of the amount of TS, probably due to the increased use of 3'SL as a donor substrate in the reverse reaction, finally leading to an equilibrium between lactose, 3'SL, sialylated and desialylated glycans on fetuin. This equilibrium apparently was reached in 30 minutes with 500 ng TS (266 ± 4 μM 3'SL for *T. cruzi *TS, 194 ± 6 μM 3'SL for TS1b and 165 ± 7 μM 3'SL for TS1 e-1). After 20 h incubation, 50 ng TS was sufficient to reach the equilibrium. Independent of the amount of enzyme used, for all three TS applied similar final concentrations of 3'SL were obtained after 20 h incubation (Table 1).

**Table 1:** Free Sia and 3'SL production at equilibrium of the reaction.

	Neu5Ac [pmol]	3'SL [pmol]
**0 ng TS**	262 ± 46	0

**50 ng *T. cruzi *TS**	580 ± 8	12597 ± 115

**250 ng *T. cruzi *TS**	1820 ± 13	11199 ± 288

**50 ng TS1b**	415 ± 7	15378 ± 117

**250 ng TS1b**	1095 ± 129	14435 ± 2226

**50 ng TS1 e-1**	348 ± 83	12719 ± 3057

**250 ng TS1 e-1**	1186 ± 9	13655 ± 491

**only fetuin**	116 ± 8	0

Neu5Ac and 3'SL produced by TS after 20 h incubation under standard conditions were determined as described under Material and Methods. Data points are means ± standard deviations of three replicates.

The HPAEC-PAD method used allowed not only determining the TS, but also SA activity, since free Sia and 3' SL could be quantified from the same chromatogram. In standard reactions (50 ng TS, 30 min incubation time) no SA activity could be detected, both in the presence or absence of lactose as an acceptor substrate. This suggests that these TS1 variants usually need an acceptor substrate like lactose to cleave Sia from a donor substrate. However, after 20 h incubation, free Sia was detected. The quantity of Sia released was dependent on the amount of TS used (Table 1). Besides standard TS reactions with fetuin as donor and lactose as acceptor substrate, TS reactions with 2 mM 3'SL as donor and 2 mg/mL ASF as acceptor substrate were performed. In these reactions, free Sia was detected after short reaction times and after incubation for 24 h, 0.5-1 mM free Sia were produced (data not shown).

**Figure 7:**
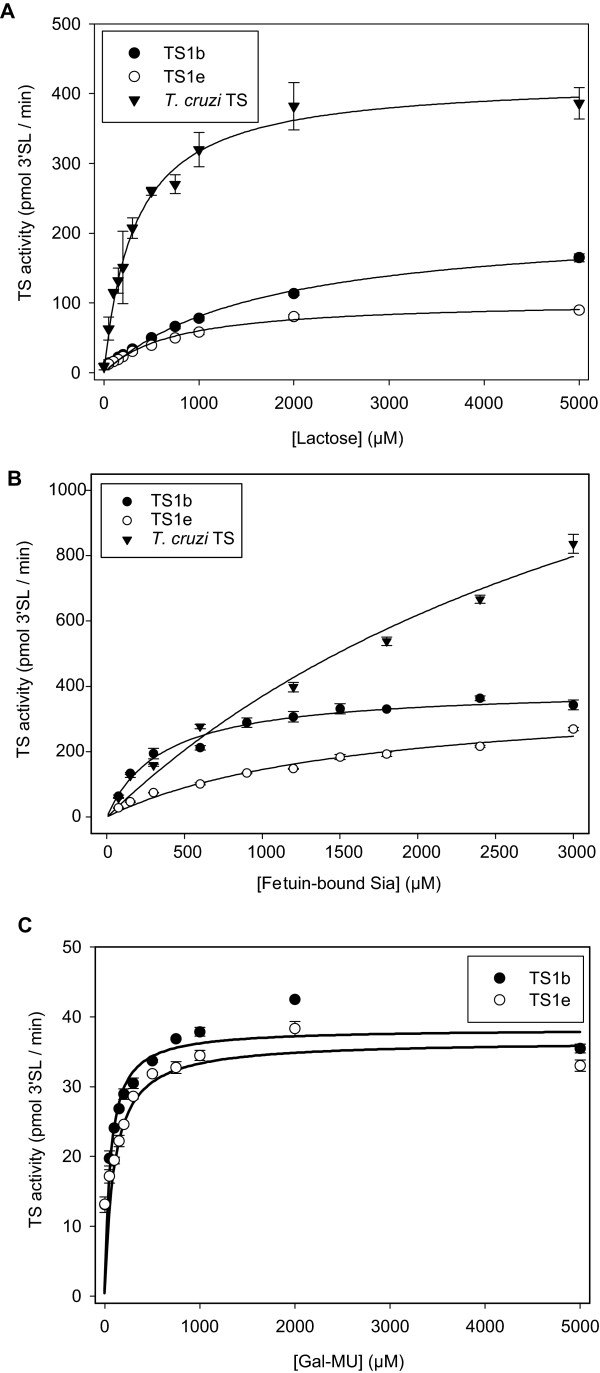
**Michaelis-Menten kinetics of TS reactions**. All reactions were started with 50 ng TS in a final volume of 50 μL and incubated for 30 min at 37°C. **(A) **600 μM fetuin-bound Sia was used as donor substrate with varying concentrations of lactose as acceptor substrate. **(B) **Varying concentrations of fetuin-bound Sia were used as donor substrate with 2 mM lactose as acceptor substrate. **(C) **Varying amounts Gal-MU were used as acceptor substrate and 50 nmol 3'SL as donor substrate. The amount of 3'SL (in the case of **(A) **and **(B)**) or 3'Sia-Gal-MU (in the case of **(C)**) produced was determined by HPAEC-PAD as described under Methods. Data points are means ± standard deviations of three replicates.

For kinetic experiments, assays were incubated for 30 minutes using 50 ng TS, since under these conditions 3'SL production was linear for all three TS. To determine the kinetic parameters for the acceptor substrates lactose (Figure 7A) or Gal-MU (Figure 7C), 100 μg fetuin (600 μM bound Sia) was used as donor substrate. The lowest K_M _for lactose was found for *T. cruzi *TS with 327 μM compared to 1683 μM for TS1b and 727 μM for TS1 e-1 (Table 2). Furthermore, *T. cruzi *TS was able to produce twice more 3'SL than TS1b and fourfold more than TS1 e-1 under these conditions.

**Table 2:** Kinetic parameters of *T. cruzi *TS, *T. congolense *TS1b and TS1 e-1

	Acceptor substrates				Donor substrates	
	
	Lactose		Gal-MU		**Fetuin-bound Sia**^**x**^	
	
	v_max_ (μmol/(min × mg TS))	K_M_ (μM)	v_max_ (μmol/(min × mg TS))	K_M_ (μM)	v_max_ (μmol/(min × mg TS))	K_M_ (μM)
**TS1b**	4.3 ± 0.1	1683 ± 101	0.77 ± 0.03	57 ± 14	7.9 ± 0.3	359 ± 45

**TS1 e-1**	2.1 ± 0.1	727 ± 48	0.72 ± 0.03	74 ± 17	7.6 ± 0.5	1617 ± 223

***T. cruzi *TS**	8.4 ± 0.3	327 ± 31	n.d.	n.d.	37.9 ± 6.0	4124 ± 985

K_M _and v_max _were calculated from Michaelis-Menten kinetics (Figure 7) by SigmaPlot. Data points are means ± standard deviations of three replicates. × Approximately 30 nmol Sia per 100 μg fetuin. Abbr.: n.d.: not determined.

To determine the kinetic parameters for the donor substrate fetuin (Figure 7B), 2 mM lactose was used as acceptor substrate. Both *T. congolense *TS1 had similar v_max_-values, whereas the v_max _for *T. cruzi *TS was about fivefold higher. Different to the K_M _of lactose, the lowest K_M _for fetuin was exhibited by TS1b with 359 μM, which is about fivefold lower compared to TS1 e-1 with 1617 μM and about 12-fold lower compared to *T. cruzi *TS with 4124 μM.

Kinetic studies with TS were also performed for the acceptor substrate Gal-MU (Table 2) and the donor substrate pNP-Neu5Ac. Almost similar K_M _and v_max_-values were found for both *T. congolense *TS1. The substrate pNP-Neu5Ac was only weakly used as a donor substrate by all three TS species. Therefore, no reliable kinetic parameters could be determined.

### Sialylation pattern of glycopeptides

**Figure 8:**
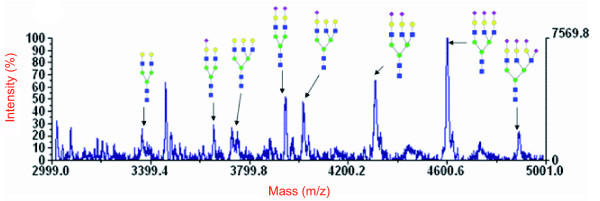
**MALDI-TOF-MS of glycopeptide 127-141 sialylation variants**. 100 μg fetuin were treated with trypsin and analyzed by MALDI-TOF-MS as described under Methods. The symbols code for the monosaccharide units is according to the nomenclature of the Consortium for Functional Glycomics (http://www.functionalglycomics.org): GlcNAc (blue squares), Man (green circles, Gal (yellow circles), Neu5Ac (purple diamonds).

Structural differences between TS1 variants may influence the enzymes preference for glycans on glycoproteins, such as fetuin. This could possibly result in different sialylation patterns on glycoproteins after incubation with TS. Fetuin contains 3 *N*-glycosylation sites and 3 *O*-glycans, which all can serve as Sia donors in TS reactions [13]. To investigate the specificity of TS towards different *N*-glycans on fetuin, we used MALDI-TOF-MS to determine the sialylation pattern of glycopeptides (GPs) from trypsin-digested fetuin after incubation with TS and lactose (Figure 8).

**Figure 9:**
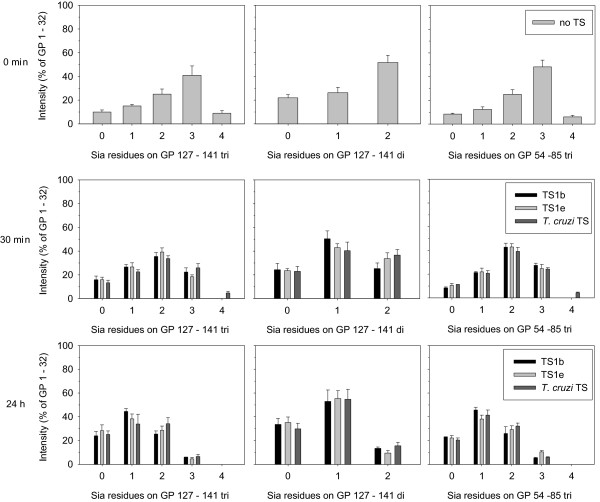
**Relative changes of the sialylation pattern of fetuin *N*-glycans**. Shown are the sialylation patterns of the glycopeptides GP 127-141 with dibranched (GP 127-141 di) or tribranched (GP 127-141 tri) glycans and of the glycopeptide GP 54-85 with tribranched glycans (GP 54-85 tri) relative to the non-sialylated GP 1 -32. 100 μg fetuin was incubated with 50 ng TS1b, TS1 e-1 or *T. cruzi *TS and 100 nmol lactose for the times indicated, trypsinized and glycopeptides analyzed by MALDI-MS as described under Methods. Data points are averages ± standard deviations of three to ten replicates.

The sialylation patterns of three glycopeptides, GP 127-141 (dibranched or tribranched), and GP 54-85 (tribranched) were determined. All 14 potential sialylation variants of these glycopeptides could be identified unambiguously and quantified from the MALDI-TOF-MS spectra. In untreated fetuin most branches on the three *N*-glycans investigated were sialylated, whereas upon treatment with TS and lactose after 30 minutes a clear shift towards incompletely sialylated glycans was observed. After 24 h TS incubation the relative amounts of unsialylated glycans was further increased and monosialylated glycans represented the most prominent species on both, di- and tribranched glycans (Figure 9).

### Sialylation of glycoproteins

**Figure 10:**
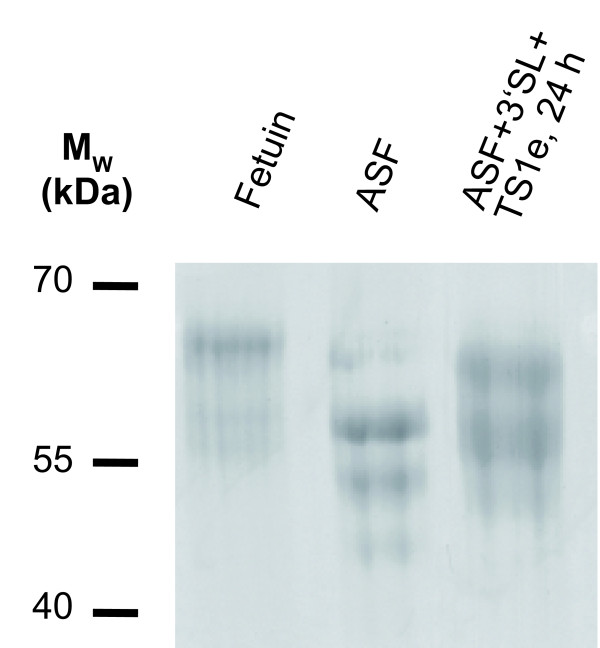
**SDS-PAGE of resialylated fetuin**. ASF (45 to 58 kDa): *Vibrio cholera *sialidase-treated fetuin (58 to 65 kDa). For resialylation ASF was treated with TS1 e-1 and 3'SL for 24 h. 2 μg protein per lane were run on a 12% polyacrylamide gel. Abbr.: M_W_: molecular weight

As described above, *T. congolense *TS1b and TS1 e-1 readily used fetuin as donor substrate for the production of 3'SL. However, long-term TS reactions or experiments with higher amounts of TS had suggested that the reverse reaction also takes place. Therefore, we investigated whether *T. congolense *TS1b and TS1 e-1 can restore sialylated glycans on *Vibrio cholerae *sialidase-treated fetuin (ASF) as model glycoprotein. Resialylation experiments were performed with 100 μg ASF as acceptor and 100 nmol 3'SL as donor substrate as well as 50 ng TS1 in 50 μL to start the TS reaction and were incubated up to 24 h. This resialylation partially reversed the shift in electrophoretic mobility in SDS-PAGE observed for sialidase-treated fetuin (Figure 10). Also by MALDI-MS of glycopeptides, the sialylation of unsialylated glycans was confirmed (data not shown).

**Figure 11:**
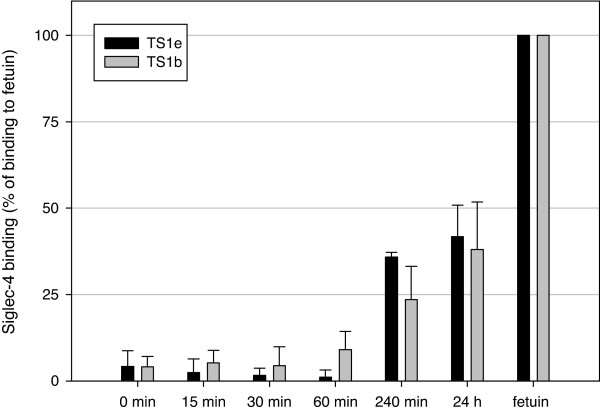
**Binding of Siglec-4 to resialylated fetuin**. *Vibrio cholera *sialidase-treated fetuin (ASF) was treated with TS1 e-1 and 3'-SL for the times indicated as described under Material and Methods. Siglec-4 binding relative to untreated fetuin is shown. Data points are means ± standard deviations of three independent determinations.

Furthermore, we addressed the question of whether through this reaction recognition sites for siglecs can be restored. For this purpose, TS-treated ASF was immobilized to a microtitre plate and used as target for Siglec-4, which preferentially binds α2,3-linked Sia. Under these conditions robust Siglec-4 binding was observed of ASF that had been treated with TS for 4 h. A prolonged (up to 24 h) TS reaction only led to little further increase reaching 40% of binding levels observed with native fetuin (Figure 11).

## Discussion

In 2003 Tiralongo et al. published a partial sequence for *T. congolense *TS1 [9]. Our approach to obtain the full length sequence of TS1 led to the discovery of 11 variants of this gene with an overall sequence identity of 96.3% in the genome of this parasite. The previously published partial TS1 sequence, which had been assembled from 47 independent PCR reactions, turned out to represent a mixture of fragments from the 11 TS1 forms identified in this study. Therefore, it is likely that that sequence doesn't exist in nature.

Similar TS-like gene families occur also in other trypanosomes. In *T. brucei *the situation appears to be less complex, since only 8 TS closely related genes have been identified [3] and these differences lead to 6 amino acid mutations. The largest TS gene family has been found in *T. cruzi*. Its 140 members fall into three different groups [14]. Blood stream trypomastigotes express two of these groups, one with TS activity and the other only with lectin activity. A third group has TS activity and is expressed by epimastigotes. At present it is unknown whether the expression of the different TS genes in African trypanosomes is also stage-dependent.

*T. congolense *TS1 shares about 57% identical amino acids with *T. brucei *TS and 48% sequence identity with *T. cruzi *TS. By comparison, the American *T. rangeli *SA and *T. cruzi *TS are more closely related with approximately 70% sequence identity [15].

The homology model for *T. congolense *TS1 based on crystal structures from *T.cruzi *TS and *T. rangeli *SA provided (I) insight in differences in the active site and its surrounding between TS from these parasites and (II) revealed the spatial distribution of the amino acid differences between the TS1 variants.

Compared to *T. cruzi *TS only three amino acids are changed in TS1 (A325, S407 and Y408). At position 325 a proline at the corresponding position of *T. cruzi *TS appears to be relevant for full TS activity [11]. However, in both, *T. brucei *TS and in *T. congolense *TS1, position 325 is an alanine. Tiralongo et al., (2003) postulated A325 might be common to African trypanosomes and does not seem to have an effect on enzymatic activity [9]. Our data have demonstrated that an alanine at this position is well compatible with TS activity, since all active TS1 variants have A325. The exchanges at 407 and 408 may be relevant for the different substrate specificities of TS species, since this area possibly participates in the interaction with the underlying galactose.

With the full length coding sequence of TS1 available, the enzymes were expressed and characterized as recombinant proteins to address the question of whether TS1 accounts for the TS activity in the two preparations (TS-form 1 and TS-form 2) from procyclic parasite cultures [6]. The amino acid sequences of the eleven TS1 variants described here contain the peptide VVDPTVVAK present in TS-form 1. Furthermore, all recombinant TS1 variants reacted with the anti-*T. congolense *TS antibody (mAb 7/23) [6]. Based on this information, it is now clear that TS1 was present in both, TS-form1 and TS-form 2. However, two observations made with recombinant TS1 suggest that the native enzyme preparations of TS-form 1 and even more for TS-form 2 contained additional TS1-like proteins. (I) The reaction velocities with the synthetic substrates pNP-Neu5Ac (as donor) and Gal-MU (as acceptor) were much lower than with fetuin and lactose for all three TS. Tiralongo et al., 2003 [6] determined a K_M _of 500 μM for Gal-MU, which is approximately 10-fold higher than for TS1b (57 μM) and TS1 e-1 (74 μM). These differences could be due to the presence of other TS1-like enzymes in the preparations of TS-form 1 and TS-form 2. (II) Using the substrate Neu5Ac-MU no SA or TS activity could be detected for the *T. congolense *TS1 variants investigated here, whereas cleavage of this substrate indicated SA activity in preparations of TS-form 1 and TS-form 2 [6], as well as TS activity in procyclic parasites [16]. Also this apparent discrepancy can easily be explained by the presence of other TS1-like enzymes accepting Neu5Ac-MU as TS or SA substrate.

Ten of the eleven recombinant TS1 variants revealed TS activity, which is in agreement with the TS-form 1 purified by Tiralongo et al. [6]. Only TS1g, which carries a cysteine at position 144 instead of an arginine, showed very low TS but clear SA activity. This suggests that R144 is important but not essential for the transfer reaction and hinders SA activity in *T. congolense *TS1. In a previous study on *T. rangeli *SA [17] R144 has been postulated to form a hydrogen bond to the O4 of Sia. However, it remains unclear how this could relate to TS activity. Furthermore, the homology model of TS1 does not provide evidence for such a hydrogen bond for the binding site of *T. congolense *TS1.

The TS1 homology model (Figure 4B) displays also the position of amino acid exchanges in the eleven TS1 forms identified. It could be speculated that these positions are relevant determinants of antigenic sites and that these variations help to escape recognition by the immune system. However, if TS1 is expressed in the procyclic form in the insect vector, this is unlikely to be relevant. Interestingly, these exchanges are located on the same side of the protein where substrate binding occurs, which opens the possibility that these changes influence the binding specificities. The cluster of changes from position 599 to 602 and 643 could be involved in recognition of larger substrate molecules, such as glycoproteins. Positions, 407 and 408 (Figure 4B), as well as positions 375, 377 and 404 (Figure 4A) are closer to the active site in the catalytic domain, possibly contributing to interactions with substrates. It should be noted that the six amino acid differences occurring in *T. brucei *TS are not found in clusters like in *T. congolense *TS1, and furthermore, they are not even on the same side of the protein.

For a more detailed characterization of their differences in activity, we choose two TS1 variants, TS1b and TS1 e-1. The two amino acid sequences of TS1b and TS1 e-1 differ mainly in the lectin domain (Figure 4), whereby the exchange from E600 in TS1b to K600 in TS1 e-1 represents the most drastic change.

The observation that lactose is a much better acceptor substrate than Gal is in agreement with previously reported relative transfer activity data for the preparations TS-form 1 and TS-form 2 [9]. Interestingly, with fetuin as donor substrate *T. cruzi *TS, *T. congolense *TS1b and TS1 e-1 produced different amounts of 3'SL in 30 minutes under identical conditions. It is likely that these differences are due to distinct substrate specificities for the sialoglycans of fetuin. However, after 20 h, equilibriums for the reactions were attained and the three TS applied produced almost the same amounts of 3'SL. Most likely this reflects a similar equilibrium for these three TS.

Clear differences were found in the kinetic parameters of TS1b and TS1 e-1 for lactose and fetuin. Reliable kinetic parameter for 3'SL as donor substrate could not be determined, because of the inaccurate quantification of the high concentrations of 3'SL as donor substrate. Whereas TS1b has a slightly higher K_M _(1683 μM) for lactose compared to TS1 e-1 (727 μM), the opposite and more pronounced difference was observed for fetuin-bound Sia, where TS1 e-1 has approximately fivefold higher K_M _(81 μM) compared to TS1b (17 μM). In combination, this implies that the ratio of K_M _for lactose/K_M _for fetuin-bound Sia is approximately 100 for TS1b, whereas it is only 10 for TS1 e-1. By comparison, for *T. cruzi *TS the K_M _for both substrates is quite similar (326 over 206 μM) and its v_max _was fourfold higher than for the TS1 isoforms. The differences in the kinetic parameters for fetuin observed for TS1b and TS1 e-1 are possibly related to altered affinities resulting from amino acid divergences in the lectin domain of TS1b and TS1 e-1. This would suggest a mechanism linking the lectin domain to the enzymatic properties of TS.

No release of free Sia could be detected after 30 minutes of TS reactions with fetuin as donor and lactose as acceptor substrates, demonstrating the absence of SA activity. However, after 20 h incubation, free Sia was detected clearly indicating SA activity. This activity correlated with the TS amount present, implying SA activity to be a side reaction observable only in extended reactions. Interestingly, in the reverse TS reactions with 3'SL as donor substrate for sialylation of galactose residues of ASF, free Sia is detected very early in the reaction (data not shown). This suggests that the free Sia detected in extended TS reactions times with fetuin as donor substrate is mainly the product of a SA side activity of the reaction using 3'SL as substrate. Therefore, the amount of free Sia could provide indirect information on the velocity of the reverse reaction. This assumption is further supported by the fact that lower amounts of TS can led to the same amount of final 3'SL but produce less free Sia as side product (Table 1). In this context it should be noted, that TS from *T. cruzi *clearly produced more free Sia than *T. congolense *TS1b or TS1 e-1. The structural basis for this phenomenon is unclear but may be related to the kinetics of the reaction. TS have been reported to follow ping-pong bi bi kinetics [12,18,19]. It will be interesting to investigate the structure-function relationship of this phenomenon and whether this is related to the SA activity of TS1g.

The TS substrate specificities for the glycans of the donor substrate fetuin were investigated by a MALDI-TOF analysis of TS treated glycopeptides from trypsin digested fetuin, since the glycosylation of fetuin is well established [13,20-23]. Three glycopeptides coming from two of the three *N*-glycosylation sites (di- and tribranched GP 127-141, and tribranched GP 54-85) could be analyzed reliably. The predicted masses of GP 142-169 di could not be identified in any spectra. GP 54-85 di and GP 142-169 tri differed only in one Da, which were not resolved by the equipment available and were excluded from the analysis. The peptide containing the three *O*-glycosylation sites could not be detected due to its high mass. But it is important to note, that TS1 clearly utilizes sialylated *O*-glycans as donor substrates as indicated by a rapid unmasking of peanut agglutinin recognition sites (data not shown).

Only minor differences in the sialylation pattern of fetuin GPs were observed using the different TS species. In summary, these were too small to draw a conclusion that these TS differ in their substrate specificities for fetuin glycans. In general, the TS applied cleaved Sia from *N*-glycans of glycopeptides investigated, but also transferred Sia back to these *N*-glycans. *N*-glycans that carried three and four Sia molecules in the case of the tribranched *N*-glycans as well as *N*-glycans that carried two Sia molecules in the case of dibranched *N*-glycans before TS incubation, were reduced to predominantly 0-2 Sia molecules in the case of tribranched and 0-1 Sia molecule in the case of dibranched *N*-glycans after 24 h TS incubation.

Whereas in the reactions discussed above Sia was transferred from fetuin to synthesize 3'SL, we could also show that *T. congolense *TS1 as well as *T. cruzi *TS transfer Sia in the reverse direction from 3'SL to glycoproteins. Furthermore, the TS reaction restores binding of Sia-binding proteins, such as Siglec-4. Due to the reversibility of the reaction, a complete resialylation of an acceptor substrate applying TS cannot be expected under these conditions. Nevertheless, differences in the kinetic parameters as shown for two of the eleven *T. congolense *TS1 variants could be used in kinetically controlled reactions to optimize the TS reaction to one or the other product, making the TS1 variants interesting tools for biotechnological applications. Thus, TS1 can be utilized to transfer Sia in α2,3-linkage on biologically relevant glycoproteins containing terminal galactose as Sia acceptor.

## Conclusions

For the first time, full length TS from the African parasite *T. congolense *has been cloned and sequenced, opening new perspectives for investigations on the biological role of these enzymes in the pursuit of a cure for Nagana and sleeping sickness. Eleven *T. congolense *TS1 variants were identified and expressed as recombinant proteins. The eleven TS1 differ in 25 of 702 amino acid positions and a structural model revealed that these variations occur in three clusters on the side of the protein that is open to substrate binding. Ten of these TS1 variants share predominantly TS and little SA activity. Only one, TS1g, has much lower TS but increased SA activity, probably due to an exchange of an arginine to a cysteine at position 144. Interestingly, the kinetic parameters of two characterized TS1 variants reveal subtle differences in substrate specificities. However, these did not lead to major differences in the sialylation pattern of *N*-glycans on fetuin after treatment with different TS variants. Finally a proof of principle has been provided that these TS can be used to sialylate glycoconjugates to create binding sites for Sia-binding proteins like Siglec-4.

It will be interesting to investigate the expression patterns of TS1 variants in the parasite's life cycle in future investigations addressing their importance for the manifestation of midgut colonization and maturation in the tsetse vector with possible implications for the transmission to the mammalian host.

## Methods

### Materials

Complete Mini, EDTA free protease-inhibitor tablets and *Vibrio cholerae *sialidase were purchased from Roche Diagnostics, Mannheim, Germany. *Pfu *DNA polymerase and restriction enzymes *Bam*HI and *Spe*I, isopropyl β-D-1-thiogalacto-pyranoside (IPTG), PageBlue and molecular weight marker (PageRuler) were from Fermentas, St. Leon-Rot, Germany. Trypsin was from Promega, Mannheim, Germany, 2,5-Dihydroxybenzoic acid from Bruker Daltonics, Billerica, USA. Ultrafiltration units Vivaspin6 and VivaCell250 were from Sartorius, Göttingen, Germany. BCA Protein Assay Kit was purchased from Thermo Scientific Pierce, Rockford, USA. Anti-SNAP-tag rabbit polyclonal antibody was from GenScript, Piscataway, USA. Anti-*Strep*-tag rabbit polyclonal antibody, StrepTactin beads and buffers were purchased from IBA, Göttingen, Germany and hygromycin from PAA, Pasching, Austria. 2'-(4-Methylumbelliferyl)-α-D-*N*-acetylneuraminic acid sodium salt hydrate (MU-Neu5Ac), 4-methylumbelliferyl ß-D-galactoside (MU-Gal), 4-methylumbelliferone (MU), glucoronic acid, Ex-cell^® ^CD CHO media, fetuin and PEI transfection reagent were purchased from Sigma-Aldrich, Munich, Germany. X-ray film, enhanced chemiluminescence system, Ni-NTA and Q-Sepharose FF were purchased from GE Healthcare, Munich, polyvinylidene difluoride membranes and ZipTips from Millipore, Schwalbach, Germany.

### Cloning and expression of recombinant TS1

The published partial sequences of *Trypanosoma congolense *TS1 [Genebank: AJ535487.1] [6,9] was used as starting query for searching the *T. congolense *genomic database for pathogen genomics at the WTSI (http://www.sanger.ac.uk/). Based on the obtained sequence fragments, an open reading frame encoding TS1 was assembled. Based on this, primers were designed to amplify TS1 from *T. congolense *(strain STIB 249) genomic DNA [9] using *Pfu *DNA polymerase in a nested PCR reaction leaving out the N-terminal signal peptide sequence and the C-terminal GPI anchor attachment site. Both outside primers (forward ATG CGG CCG GTG AAT TGT TAN and reverse CAT CAG CAC ATG CAC GAG CAN) were degenerate at the 3' end, whereas the internal primers (forward CGA CTA GTC AGT GCT GCG ACC ACA TGC AN and reverse CGG GAT CCG TCG CTC CCA GGC ACA CGA AN) were designed to introduce *Spe*1 and *Bam*HI restriction sites, respectively. These restriction sites were used to ligate the PCR-products in frame into a modified pDEF [24] vector (pDEF-T3C/SNAPstrep) providing in frame a Transin cleavable signal peptide [25], a 3C-protease cleavage site [26] followed by SNAP (Covalys, Witterswil, Switzerland) and *Strep *(IBA, Göttingen, Germany) tags. The pDEF-T3C/SNAPstrep was obtained as follows: The coding sequence for the Transin signal peptide has been introduced into pcDNA 3 Amp *Strep-tag *[27] using the *Hind*III/*Bam*HI-digested linker obtained by hybridization of the following oligonucleotides: sense 5'-CGAAGCTTATGAAAGGGCTCCCAGTCCTGCTGTGGCTGTGTACGGCTGTGTGCTC

ATCCTACCCATTGCATGGCAGTGAAGAAGATGCTGGCATGGAGACTAGTGGATCCCG

and antisense primer 5'-

CGGGATCCACTAGTCTCCATGCCAGCATCTTCTTCACTGCCATGCAATGGGTAGG

ATGAGCACACACAGCCGTACACAGCCACAGCAGGACTGGGAGCCCTTTCATAAGCTTCG.

This Transin linker introduced a unique *Spe*I restriction site. The coding sequence of the hAGT protein (SNAP) was amplified using the pSNAP-tag^® ^(T7) vector (NEB, Ipswich, MA, USA) as a template and subcloned in frame into *Bam*HI/*Eco*RI digested pcDNA3 Amp Transin *Strep*-tag providing pcDNA3-T3C/SNAPstrep. The primers used for amplification were: sense 5'-CGGGATCCCTGGAGGTGCTGTTCCAGGGCCCCATGGACAAAGACTGCGAAATGAAGCG-3' including the coding sequence for the 3C protease recognition site of the human rhinovirus HRV 3C (LEVLFQGP, underlined) and antisense 5'- CGGAATTCACCCAGCCCAGGCTTGCCCAGA.

CHO_Lec1 _cells were used for TS1 expression due to their ability to express only high-mannose glycans, since these cells are lacking *N*-acetyl glucosaminyltransferase I [28]. Transfection of CHO_Lec1 _cells grown in αMEM supplemented with 10% fetal calf serum at 37°C, 5% CO_2 _was accomplished with polyethylenimine (PEI) transfection reagent following the manufacturer's instructions. 24 h after transfection, cells were passaged into 96-well plates in a selection media of αMEM containing varying amounts of hygromycin, ranging from 400 μg/mL to 1000 μg/mL. Expression of recombinant TS1 (120 kDa including SNAP and *Strep *tags) was tested by analyzing cell culture supernatant using Western blots with anti TS1 monoclonal antibody (mAb 7/23) as primary antibody [9]. The presence of SNAP and *Strep *tags was confirmed using anti-SNAP-tag rabbit polyclonal antibody and anti-*Strep*-tag rabbit polyclonal antibody respectively in Western blots analysis. Selected cells were then adapted to Ex-cell^® ^CD CHO media supplemented with 8 mM L-glutamine.

### Purification of *Trypanosoma congolense *trans-sialidase

The harvested tissue culture supernatant was supplemented with 10 mM Tris/HCl, pH 7.5, 1 mM EDTA, 1 mM DTT and 0.02% sodium azide (all final concentrations) and centrifuged at 125,000 × g for 1 h. The cleared supernatant was then concentrated 100-fold by ultrafiltration (100 kDa cut off). Buffer was exchanged twice using 250 mL 100 mM Tris-Cl, pH 8.0, 150 mM NaCl, 1 mM EDTA (buffer A) in the same ultrafiltration unit and concentrated to a total volume of 10 mL for 1 L tissue culture supernatant. This was further clarified by centrifugation at 21,000 × g for 30 minutes before applying on a column of 1 mL *Strep*Tactin^® ^beads equilibrated with buffer A. After loading the column was washed with 5 column volumes wash buffer (100 mM Tris-Cl, pH 8.0, 150 mM NaCl, 1 mM EDTA) and TS1 was eluted with 3 column volumes of elution buffer (100 mM Tris-Cl, pH 8.0, 150 mM NaCl, 1 mM EDTA, 2.5 mM desthiobiotin) in fractions of 0.5 mL. The affinity purified TS1 was dialyzed (10 kDa cut off) against 10 mM phosphate, pH 7.4. Purification products were analyzed by SDS-PAGE and quantified by BCA assay with bovine serum albumin as standard.

### Expression and purification of *Trypanosoma cruzi *trans-sialidase

Recombinant *T. cruzi *TS was produced in *E. coli *M15 (pREP4) according to Agusti et al., 2004 and Neubacher et al., 2005 [29,30]. In brief, cells were grown in 1 L "terrific broth" medium overnight at 18°C. Protein expression was initiated with 0.5 mM IPTG. The cells were dissolved in 40 mL lysis buffer (50 mM phosphate, 300 mM NaCl, pH 8.0 and 0.05% Lubrol), 1 tablet protease-inhibitor (Complete Mini, EDTA free) and 1 spatula tip of lysozyme were before incubation of 30 minutes at 4°C. The cells were disrupted by 5 cycles of sonification on ice. Cells debris was removed by centrifugation at 40,000 × g for 60 minutes at 4°C and the supernatant was filtered using a 0.2 μm pore size filter. 20 mM imidazol was added before application on 0.5 mL Ni-NTA beads. Target proteins were eluted in the same buffer and 250 mM imidazole. The eluted protein was dialyzed against 20 mM Tris, 30 mM NaCl, pH 8.0 and further purified using a Q-Sepharose FF column in the same buffer with a linear gradient up to 1 M NaCl. The activity of the purified protein was tested by a sialidase activity assay using MU-Neu5Ac as donor substrate and lactose as acceptor substrate as described below.

### *Vibrio cholerae *sialidase treatment of fetuin

Asialofetuin (ASF) was prepared from fetuin by *Vibrio cholerae *sialidase (VCS) treatment as described [31]. In brief, fetuin was digested with VCS in 50 mM sodium acetate, 9 mM CaCl_2_, pH 5.5 overnight at 37°C in a dialysis bag against the same buffer and afterwards against distilled water. Sialylated fetuin and sialidase was separated from ASF by anion exchange chromatography using Q-Sepharose. The proteins were eluted by a linear gradient from 0 to 1 M NaCl in 10 mM Tris, pH 7.4. Collected fractions were assayed for protein at 280 nm and for SA activity with MU-Neu5Ac acid as substrate as described below. The fractions containing ASF but no SA activity were pooled and the buffer was exchanged against 10 mM phosphate, pH 7.4 using VivaSpin6 ultrafiltration units (10 kDa cut off).

### Sialidase activity assay

Sialidase activity (+/- lactose) was determined by applying a microtitre plate assay detecting free 4-methylumbelliferone (MU) released from Neu5Ac-MU [6]. In brief, 50 μL sample were incubated with 1 mM Neu5Ac-MU (final concentration) in a black 96-well microtitre plate. To determine *T. cruzi *TS 1 mM lactose was added as acceptor substrate. The plate was centrifuged for 1 minute at 1,000 × g and incubated for 30 minutes at room temperature in the dark. The reactions were stopped with 200 μL 100 mM glycine, pH 10 and the fluorescence intensities were measured at 355 nm excitation and 460 nm emission using a fluorimeter (Ascent Fluoroscan).

### Trans-sialidase reactions

The principle of this assay is based on the quantification of sialylated oligosaccharides by high performance anion-exchange chromatography with pulsed amperometric detection (HPAEC-PAD) as described below. In these reactions either fetuin is used as donor substrate, e.g. lactose as acceptor or 3'-sialyllactose (3'SL) is used as donor substrate with ASF as acceptor. A final volume of 50 μL 10 mM phosphate buffer were used for all TS reactions. Stock solutions of donor and acceptor substrate were mixed in 40 μL buffer (10 mM Tris/HCl, pH 7.5) and the reactions were started with 10 μL TS (50 ng in standard reactions) and incubated at 37°C. The reactions were stopped with 200 μL ice-cold acetone containing 28 μM glucuronic acid and incubated overnight at -20°C. After centrifugation for 15 minutes at 20,000 × g and 4°C, 225 μL supernatant were removed and both, protein pellets and supernatants were lyophilized.

The dried supernatant of the acetone precipitation was dissolved in 125 μL H_2_O for HPAEC-PAD, which was carried out by using a DX600 system (Dionex, Sunnyvale, CA, USA) with an electrochemical detector (ED50), a gradient pump (GP50) and an autosampler (AS50). Carbohydrates were separated by HPAEC on a CarboPAC PA1 (4 × 250 mm) analytical column (Dionex) together with a guard column (4 × 50 mm) using a constant flow rate of 1 mL/min. Sample volumes of 25 μL were injected and the chromatography was performed as follows: 100 mM NaOH for 2 min, followed by 100 mM NaOH/100 mM NaOAc for 22 min. The column was regenerated by washing for 5 min with 100 mM NaOH/500 mM NaOAc, followed by 5 min with 100 mM NaOH. For PAD the typical quadruple waveform was used as described previously [32]. The Dionex software Chromeleon 6.40 SP8 was used for data acquisition and data evaluation.

### Calculation of kinetic parameters

V_max _and K_M _were calculated using the curve fit module of SigmaPlot 11 employing the Michaelis-Menten equation v = v_max _×c_s_/(c_s _+ K_M_).

### Siglec-4 binding assay

Murine Siglec-4_d1-3_-Fc was purified by protein-A affinity chromatography from tissue culture supernatants of stably transfected CHO Lec3.2.8.1 as described before [33]. The protein solution was dialyzed against 10 mM phosphate buffer pH 7.4, sterile filtered and stored at 4°C. Binding assays with Siglec-4 were performed as described previously [33]. In brief, 4 μg/mL fetuin, ASF or TS-treated fetuin were immobilized in microtitre plates and binding of serially diluted Siglec-4_d1-3_-Fc (8 dilutions starting with 16 μg/mL) was determined using alkaline phosphatase-labeled anti-Fc antibodies. The concentrations sufficient for 50% binding (relative to Siglec-4_d1-3_-Fc binding to fetuin) were determined from corresponding binding curves. At least three independent titrations were performed.

### SDS-PAGE and Western Blot analysis

Samples were separated by SDS-PAGE (MiniProtean III; Bio-Rad, München, Germany) according to Laemmli [34] and stained with PageBlue.

For Western blot analysis, samples were transferred onto polyvinylidene difluoride membranes after SDS-PAGE. The membranes were blocked with 5% BSA in Tris-buffered saline (TBS) buffer containing 0.15% Tween20 (TBS-T) for 1 h. Washing of the membrane was done five times for 5 minutes each using TBS-T. Immunodetection was performed by incubating membranes with a primary antibody diluted in blocking buffer overnight at 4°C. The following antibodies were used: anti-*T. congolense *TS mAb 7/23 (1:1000) and rabbit anti-*Strep*-tag (1:1000). Following four washes with TBS-T of 10 minutes each, the membranes were incubated with a secondary antibody conjugated to horseradish peroxidase for 2 h at room temperature. After washing four times with TBS-T, blots were developed with the enhanced chemiluminescence system using X-ray film.

### Matrix-assisted laser desorption ionisation-time of flight mass spectrometry (MALDI-TOF-MS)

TS reactions were carried out with 50 ng TS, 100 nmol lactose and 100 μg fetuin as described above. The dried protein pellets after TS reaction were dissolved in 200 μL 50 mM ammonium hydrogen carbonate, pH 7.8 and 1.6 μL 45 mM DTT were added. After 30 minutes incubation at 50°C, 1.6 μL 100 mM IAA were added and further incubated for 30 minutes at 37°C. The tryptic digestion was started with 2 μg trypsin dissolved in 1 μL 50 mM acetic acid and incubated overnight at 37°C and stored at -20°C.

5 μL of the tryptic digest were mixed with 5 μL H_2_O and 1 μL 1% trifluoroacetic acid and were directly mixed with matrix and 1 μL was applied to the MALDI-TOF-MS target plate. The remaining 9 μL were desalted using C18-reversed phase pipette tips (ZipTip). Peptides were eluted with 3 × 100 μL 12% and 30% acetonitrile in H_2_O and lyophilized. 2,5-Dihydroxybenzoic acid in 0.1% trifluoroacetic acid was used as MALDI matrix. The dried peptides were directly dissolved in 10 μL DHB solution and 1 μL of the mixture were spotted to the target plate for crystallization.

The mass spectrometry was performed using a Voyager DE Pro MALDI-TOF N_2_-Laser with a wavelength of 337 nm (Applied Biosystems, Foster City, USA). All spectra were measured in the linear detector mode. Laser intensities and the number of records per spectrum were varied manually.

Voyager software was used for data acquisition and peak detection. For quantification the peak intensity of each peptide was determined relative to the non-sialylated glycopeptide 1-32 (3459.66 Da) as internal standard. This peptide is detected in all spectra of the sialylation variants of the glycopeptides and is not changed by TS reaction (Figure 8). All measurements were repeated for at least three times.

### Homology Modeling

A homology model of *T. congolense *TS1 e-1 was calculated using the software Yasara 10.11.8 [35-40]. The crystal structure of *T. cruzi *TS (UniProt: Q26964; PDB entry: 3B69), previously reported by Buschiazzo et al., 2002 [12] was used as the template structure. A benzoylated *N*-acetylneuraminic acid derivative used as a ligand for *T. cruzi *TS in the template structure was kept in the binding site during the calculation of the homology model.

The following parameters of the Yasara homology modeling module were modified manually from the default settings of the program: Modeling speed: slow, PsiBLASTs: 6, EValue Max: 0.5, Templates total: 1, Templates SameSeq: 1, OligoState: 4, alignments: 10, LoopSamples: 50, TermExtension:10.

## Authors' contributions

HKB carried out all the HPAEC-PAD based TS assays and its data acquisition, the Siglec-4 binding assay and SDS-PAGE to determine resialylation of asialofetuin, homology modeling, alignment of amino acid sequences, supported MALDI data acquisition and drafted the manuscript. TTG carried out *T. congolense *TS1 cloning, sequencing and expression as well as *T. cruzi *TS expression. MW performed the TS1g enzyme assays. OR participated in development of the HPAEC-PAD based TS assay. PM carried out MALDI-MS analysis and its data acquisition. ED participated in MALDI-MS analysis and its data acquisition. FD established the pDEF-Transin-TS-3C-SNAP-Strep vector construct and carried out *T. congolense *TS1 cloning. SK designed and coordinated the study and supported drafting of the manuscript. All authors read and approved the final manuscript.
